# Relationship between high dose intake of vitamin B12 and glaucoma: Evidence from NHANES 2005–2008 among United States adults

**DOI:** 10.3389/fnut.2023.1130032

**Published:** 2023-04-17

**Authors:** Zhongwei Liu, Yi Hu, Yuhan Wang, Baiwei Xu, Jiangyue Zhao, Ziyan Yu

**Affiliations:** ^1^Department of Ophthalmology, Fourth Affiliated Hospital of China Medical University, Shenyang, China; ^2^Eye Hospital of China Medical University, Shenyang, China; ^3^Key Lens Research Laboratory of Liaoning Province, Shenyang, China

**Keywords:** vitamin B12, glaucoma, national health and nutrition examination survey, nutrition epidemiology, cross-sectional study

## Abstract

**Objective:**

Glaucoma has currently become the second leading cause of blindness in the world. Serum vitamin B12 level has been found to be involved in the development and progression of glaucoma. We performed the present study to confirm this association.

**Methods:**

This cross-sectional study included 594 participants aged 40 years and older in the National Health and Nutrition Examination Survey (NHANES) from 2005 to 2008. Retinal imaging was performed using the Ophthalmic Digital Imaging system (Retinography) to assess the retina for the presence of features of glaucomatous lesions. Logistic regression models were used to assess the association between dietary vitamin intake and glaucoma.

**Results:**

After screening, 594 subjects were finally included. Among all vitamin intakes, we observed significant differences between the two groups for vitamin B12 intake (5.93 vs. 4.77 mg, *p* = 0.033). According to the logistic regression results, the intake of vitamin B12 was significantly positively associated with glaucoma (model 1: OR = 1.078, 95% CI = 1.019–1.141; model 2: OR = 1.092, 95% CI = 1.031–1.158; model 3: OR = 1.092, 95% CI = 1.029–1.158). After performing a quantile regression, we observed a significant positive association between vitamin B12 intake and incident glaucoma in the fourth quartile (model 1: OR = 1.133, 95% CI = 1.060–1.210; model 2: OR = 1.141, 95% CI = 1.072–1.215; model 3: OR = 1.146, 95% CI = 1.071–1.226).

**Conclusions:**

Therefore, the above results, high-dose intake of vitamin B12 may promote the development of glaucoma.

## 1. Introduction

Glaucoma, a neurodegenerative disease, is the second leading cause of irreversible blindness, with a worldwide prevalence of 3.5% among people aged 40 to 80 years (1). With an increasing proportion of the elderly population, 111.8 million people are expected to have glaucoma by 2040 (2). The common types of glaucoma include primary open-angle glaucoma (POAG), primary closed-angle glaucoma (PCAG) and normal tension glaucoma (NTG). Commonalities between all types of glaucoma result in damage to the optic nerve, apoptosis of retinal optic ganglion cells and visual field.

defects (3–7). Retinal ganglion cell apoptosis may be the result of impaired blood supply to the head of the optic ganglion or direct toxic effects of multiple cytotoxic substances (8–10). Glaucoma is a multi-factorial disorder, and a strong association has been found between increasing age and sex and disease progression, while other factors including hypertension, genetic variation, and other environmental risk factors may also affect it (11–13). Vascular theory and mechanical theory are the two main mechanisms of glaucoma pathogenesis. For mechanical reasons, high intraocular pressure can damage ganglion cell axons (14). The vascular theory suggests that increased intraocular pressure and other risk factors contribute to insufficient blood flow to the eyes, which can cause damage to the optic nerve (15). The precise mechanism of glaucoma remains to be determined.

Vitamin B12 (cobalamin) therapy can reduce oxidative stress damage and inflammation levels of the nervous system, and it can promote the regulation of the antiviral activity and immune system, especially when combined with folic acid (16–22). Vitamin B12 (cobalamin) deficiency is the only vitamin deficiency definitively associated with optic neuropathy characterized by slow-progressing optic atrophy (23). Recent study indicates that vitamin B12 can alleviate COVID-19 symptoms, through its analgesic function and role in neuromuscular disorders (24). A cross-sectional study showed, that vitamin B12 intake was positively correlated with plasma concentration (25). Previous prospective studies have evaluated the correlation of B vitamin intake with risk of exfoliation glaucoma or exfoliation glaucoma suspect (EG/EGS) risk. Until now, there has still been conflicting results in different studies. Several authors found that vitamin B12 intake was not correlated with EG/EGS risk in different types of glaucoma (26–29). Some studies demonstrated that serum vitamin B12 levels are decreased in glaucoma patients (6, 12), but others found them to be elevated in NTG, POAG and EXG (7, 8, 11). A meta analysis demonstrated that high-dose intake of vitamins A and B, but not vitamins C, D, or E, was associated with a low prevalence of glaucoma (30, 31).

Overall, the sample size of the glaucoma group in the above studies was small (ranging from approximately 15 to 290), and the proper dose of vitamin B12 intake for glaucoma remains inconclusive. Therefore, we conducted the present study on the basis of data from national health and nutrition examination survey (NHANES) 2005–2008 aiming to further identify the evidence provided for the appropriate dose of vitamin B12 nutritional therapy for glaucoma.

## 2. Materials and methods

### 2.1. Data source and subject selection

This study is based on data from NHANES 2005–2008. NHANES is a large nationwide cross-sectional study performed by the national center for health statistics (NCHS). NHANES subjects were all U.S. masses randomly selected on the basis of a sampling design, who underwent universal examination and signed an informed con-sent form. The NCHS research ethics review board approved the survey protocol for NHANES (32).

### 2.2. Defining criteria for glaucoma

Participants aged 40 years or older underwent binocular non-mydriatic fundus photography in the Mobile Examination Center (MEC) using the Canon Non-Mydriatic Retinal Camera CR6-45NM. Digital images were graded at the University of Wisconsin. The optic disc images were classified into 4 severity levels, no, possible, probable, definite (33). To better assess the potential risk of vitamin intake on the occurrence of glaucoma, in this study, “possible, probable and definite” were all considered to have glaucoma or a greater likelihood of developing glaucoma, and thus these subjects were all defined as having glaucoma.

### 2.3. Determination of vitamin intake and daily energy intake

Dietary data were collected in the in-person interview using the automated multiple pass method (AMPM). The AMPM is a USDA’ dietary data collection instrument and a fully computerized recall method. The NHANES (MEC) provided a set of measuring guides that facilitated participants’ ability to describe the amount of foods they had ingested (34, 35). NHANES performed dietary data statistics for two consecutive days, and we considered the mean of two daily dietary data for each subject as the final dietary intake data in an effort to obtain an outcome that more closely approximated the true level of life. Our study included all vitamin data that appeared in NHANES 2005–2008.

### 2.4. Assessment of covariates

Sociodemographic variables including age, race/ethnicity, sex and educational level were obtained by computer-assisted in-person interview (36). Daily intake of calories and diabetes mellitus were defined by subject’s self-report (34, 35, 37).

### 2.5. Statistical analysis

All statistical analyses were performed using SAS 9.4 and R software 4.1.3. NHANES uses a stratified, multistage sampling method, so we incorporated sampling weights and strata, and sampling units in our statistical analysis to account for the complex sampling design. Continuous variables were presented with mean and standard error (SE), and categorical variables were presented with percentage and SE; the chi-square test or T-test was used to compare patients’ demographic characteristics. Logistic regression models were used to determine the association of vitamin intake with the presence of glaucoma. Model 1 was adjusted by age, race, sex, and educational level. Model 2 = model 1 and adjusted by daily energy intake. Model 3 = model 2 and adjusted by diabetes mellitus. Since a significant association between vitamin B12 and glaucoma was observed, we further performed quantile regression between vitamin B12 and glaucoma. In response to the above logistic regression results, we have created additional forest plots to show them more clearly.

## 3. Results

### 3.1. Description of baseline information of the study sample

**Figure 1:**
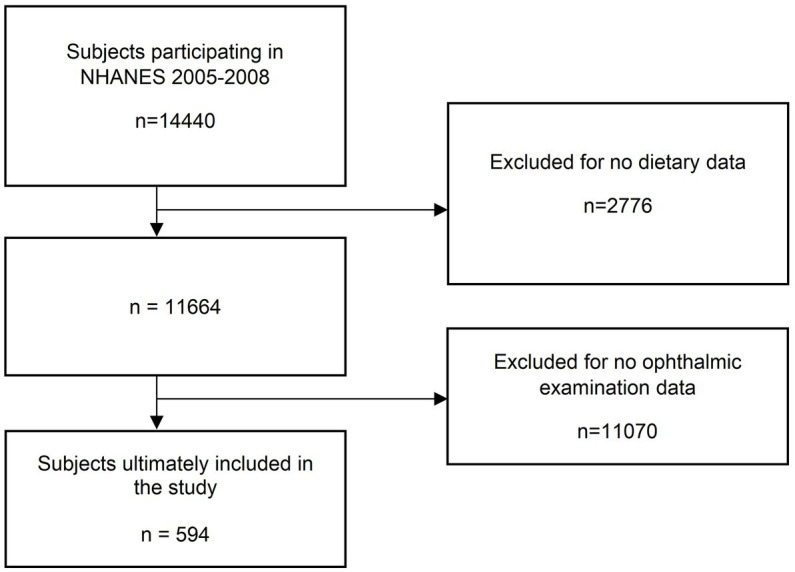
Screening process of the included studies.

On the basis of the study design of NHANES, we selected a total of 14,440 subjects for inclusion in this study. After screening, 594 subjects were finally included, and 13,846 subjects were excluded because of missing dietary data or ophthalmological examination data. The detailed flow is shown in Figure 1. Table 1 shows the demographic data as well as other characteristic data of the participants with and without glaucoma. Among the tested population, the number of subjects with or suspected glaucoma accounted for 41.9% after weighting. Of all covariates, only age differed significantly between the two groups (55.66 vs. 63.29 years, *p* < 0.001). Among all vitamin intakes, we observed significant differences between the two groups for three vitamins, retinol (474.49 vs. 401.42 mg, *p* = 0.014), vitamin A (704.61 vs. 605.62 mg, *p* = 0.0040), and vitamin B12 (5.93 vs. 4.77 mg, *p* = 0.033).

**Table 1:** Baseline information for the study sample.

Variables		Glaucoma (+)	Glaucoma (−)	*p*-Value
Continuous variables, mean (SE)				
Age (years)		63.29 (1.07)	55.66 (0.81)	<0.001
Vitamin E (mg)		7.23 (0.43)	7.31 (0.38)	0.85
Retinol (mg)		474.49 (27.83)	401.42 (16.60)	0.014
Vitamin A (mg)		704.61 (31.88)	605.62 (32.88)	0.0040
Vitamin B1 (μg)		1.60 (0.067)	1.56 (0.062)	0.54
Vitamin B2 (mg)		2.29 (0.11)	2.15 (0.082)	0.32
Niacin (mg)		23.37 (0.98)	23.64 (0.74)	0.78
Vitamin B6 (mg)		2.01 (0.085)	1.90 (0.065)	0.17
Total folate (mg)		386.13 (16.94)	402.037 (18.46)	0.20
Vitamin B12 (mg)		5.93 (0.52)	4.77 (0.19)	0.033
Vitamin C (mg)		92.72 (5.58)	86.19 (5.38)	0.25
Vitamin K (mg)		108.12 (10.64)	112.26 (14.079)	0.70
Energy (kcal)		1947.51 (73.27)	2053.12 (61.18)	0.16
Category variables, (%)				
Glaucoma		41.90 (2.10)	58.10 (2.10)	
Gender	Male	52.70 (4.50)	45.10 (3.90)	0.25
	Female	47.30 (4.50)	54.90 (3.90)	
Race	Mexican American	4.40 (1.10)	7.70 (1.20)	0.36
	Other Hispanic	1.50 (0.70)	3.70 (1.20)	
	Non-Hispanic White	69.80 (4.60)	65.20 (4.40)	
	Non-Hispanic Black	18.00 (3.00)	17.70 (2.90)	
	Other Race—Including Multi-Racial	6.30 (2.90)	5.60 (1.90)	
Education Level	Less Than 9th Grade	6.40 (2.00)	4.40 (1.40)	0.85
	9–11th Grade (Includes 12th grade with no diploma)	13.00 (2.50)	11.30 (1.80)	
	High School Grad/GED or Equivalent	23.30 (3.60)	25.40 (2.90)	
	Some College or AA degree	31.50 (3.80)	33.10 (3.10)	
	College Graduate or above	25.80 (4.40)	25.80 (3.80)	
Diabetes mellitus	(+)	19.30 (3.20)	12.30 (2.50)	0.086
	(−)	80.70 (3.20)	87.70 (2.50)	

### 3.2. Association between the intake of retinol, vitamin a, and vitamin B12 and the presence of glaucoma

**Figure 2:**
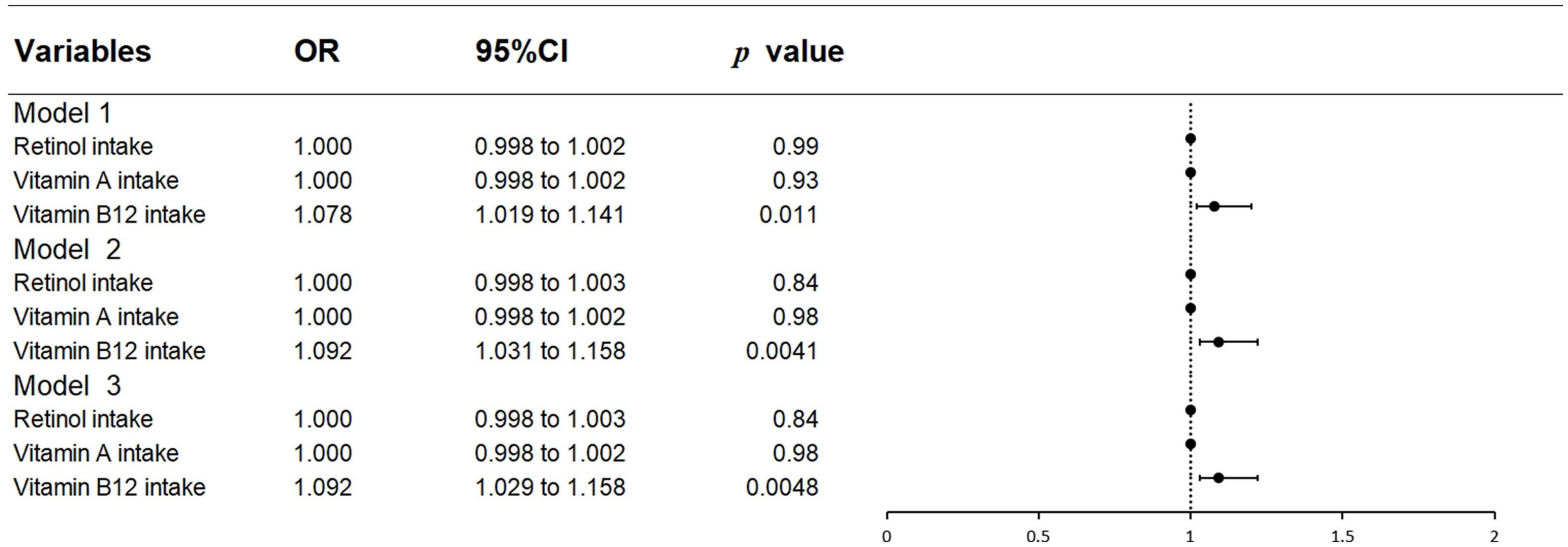
Forest plot of logistic regression results.

Table 2 and Figure 2 show the associations that existed between the intake of retinol, vitamin A and, vitamin B12 and glaucoma as addressed by multivariate logistic regression models. A significant positive association between vitamin B12 intake and incident glaucoma was shown in all models (model 1: OR = 1.078, 95% CI = 1.019–1.141; model 2: OR = 1.092, 95%CI = 1.031–1.158; model 3: OR = 1.092, 95% CI = 1.029–1.158). No significant association with glaucoma was observed for the intakes of retinol and vitamin A.

**Table 2:** Association between intake of retinol, vitamin A, vitamin B12 and glaucoma.

Variables	Model 1^a^ OR (95% CI)	*p*-Value	Model 2^b^ OR (95% CI)	*p*-Value	Model 3^c^ OR (95% CI)	*p*-Value
Retinol intake	1.000 (0.998–1.002)	0.99	1.000 (0.998–1.003)	0.84	1.000 (0.998–1.003)	0.84
Vitamin A intake	1.000 (0.998–1.002)	0.93	1.000 (0.998–1.002)	0.98	1.000 (0.998–1.002)	0.98
Vitamin B12 intake	1.078 (1.019–1.141)	0.011	1.092 (1.031–1.158)	0.0041	1.092 (1.029–1.158)	0.0048

a Model 1: adjusted for age, race, gender, educational level.

b Model 2: further adjusted for daily energy intake.

c Model 3: further adjusted for diabetes mellitus.

### 3.3. Relationship of different quartiles of vitamin B12 with the presence of glaucoma

**Figure 3:**
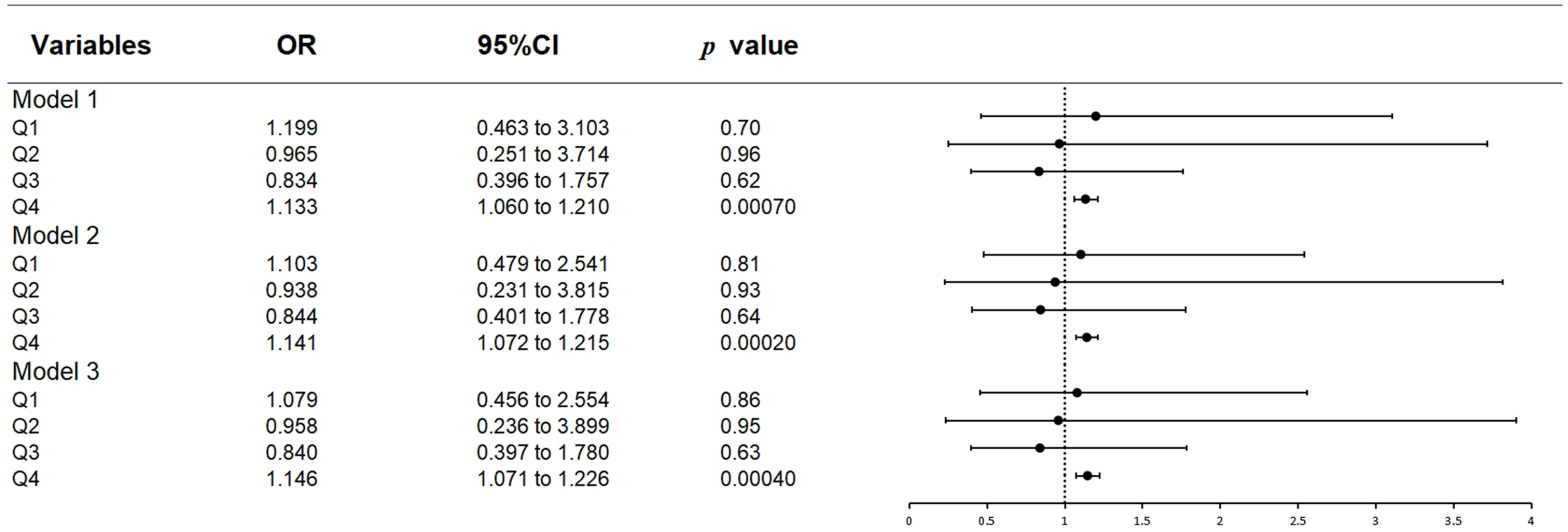
Forest plot of quantile regression results.

Table 3 and Figure 3 demonstrate the analysis of the association of different grades of vitamin B12 intake with glaucoma after dividing vitamin B12 intake into quartiles. Significant positive correlations between the fourth quartiles (Q4, high dose of vitamin B12 intake) and the prevalence of glaucoma were seen in all models (model 1: OR = 1.133, 95%CI = 1.060–1.210; model 2: OR = 1.141, 95% CI = 1.072–1.215; model 3: OR = 1.146, 95% CI = 1.071–1.226). No significant association with glaucoma was observed for the intakes of vitamin B12 in the first quartiles (Q1, low dose of vitamin B12 intake), the second quartiles (Q2, normal dose of vitamin B12 intake) and the third quartiles (Q3, normal dose of vitamin B12 intake).

**Table 3:** Association between vitamin B12 intake levels and glaucoma in different quartiles.

Variables		Model 1^a^ OR (95% CI)	*p*-Value	Model 2^b^ OR (95% CI)	*p*-Value	Model 3^c^ OR (95% CI)	*p*-Value
Vitamin B12 intake	Q1	1.199 (0.463–3.103)	0.70	1.103 (0.479–2.541)	0.81	1.079 (0.456–2.554)	0.86
	Q2	0.965 (0.251–3.714)	0.96	0.938 (0.231–3.815)	0.93	0.958 (0.236–3.899)	0.95
	Q3	0.834 (0.396–1.757)	0.62	0.844 (0.401–1.778)	0.64	0.840 (0.397–1.780)	0.63
	Q4	1.133 (1.060–1.210)	0.00070	1.141 (1.072–1.215)	0.00020	1.146 (1.071–1.226)	0.00040

a Model 1: adjusted for age, race, gender, educational level.

b Model 2: further adjusted for daily energy intake.

c Model 3: further adjusted for diabetes mellitus.

## 4. Discussion

In this study, the potential correlation between vitamin B12 intake and glaucoma was investigated by analyzing the NHANES database. Our results suggest that there is no significant correlation between normal or low doses of vitamin B12 intake and glaucoma, but that there is significant correlation between high dose intake of vitamin B12 and glaucoma.

According to past experience, the clinical consequences of multiple doses of oral vitamin B12 as a nutritional therapy for glaucoma have not been definitively studied (38). Studies have found that the main reason for high levels of serum cobalamin is the presence of potentially life-threatening diseases, and early diagnosis is often a decisive predictor (39, 40). Cobalt is a nerve agent that can cause optical neuropathy and retinopathy. Apostoli et al. injected cobalt alone intravenously, and observed optic nerve damage and loss of cochlear hair cells (41). This study, along with one by Carelli et al. exploited similarities between mitochondrial disease and cobalt-induced optic neuropathy (41, 42). Other studies have shown similar toxic effects of cobalt on the eye, such as optic nerve atrophy; however, as reported by Apostoli et al., the concentration required to produce this effect is 1/80 compared to the previous study (43). Our results showed that high-dose vitamin B12 intake may cause optic neuropathy and play a role in the development of glaucoma, consistent with these previous studies on cobalt induced visual impairment and neuropathy. There are three major pathological mechanisms underlying increased cobalamin in serum, and these mechanisms arise from any pathological factors, including: a direct increase of vitamin B12 in plasma *via* overuse or medical use; a direct increase in the level of vitamin B12 in the plasma due to release from the body *via* excessive secretion or excretion disorders; and lack of vitamin B12 levels or lack of affinity (44). Excess vitamin B12 intake when indoors is usually relatively undetected according to ANAMNEA data. In addition, long-term pastoral use of vitamin B12 may lead to the formation of an autoantibody to TK II, which leads to a decrease in its clearance (45, 46). A positive association has been observed between intake and plasma concentrations for vitamin B12 in physically active people (25). An increase in plasma vitamin B12 may indicate a functional deficit, with clinical results similar to vitamin B12 deficiency, which can lead to increased homocysteine levels, optic neuropathy, and more seriously, irreversible damage to the nervous system (47–54). This is presumed to be another mechanism of high doses of vitamin B12 as a risk factor for glaucoma development.

The strengths of this study included the focus on the relationship between vitamin B12 intake and glaucoma, and the relatively large sample size, but there were some limitations. First, the data of NHANES ophthalmology examination could not clearly indicate the type of glaucoma that the subject had and could not reveal the relationship between vitamin B12 and different types of glaucoma. Additionally, the diet data obtained from the self-reported recall of the subject could have had some errors.

There were also individual differences in the bioavailability of vitamin B12 in each participant, resulting in differences in serum vitamin concentrations (29). Therefore, further controlled trials or epidemiological peer studies are required to confirm the serious consequences of high doses of vitamin B12 in different types of glaucoma. Moreover, to further investigate the direct relationship between vitamin B12 and glaucoma, future research should be devoted to the analysis of serum vitamin levels. Despite the limitations, this study is valuable in light of the association between high-dose intake of vitamin B12 and glaucoma.

## 5. Conclusion

High-dose vitamin B12 intake may contribute to the development of glaucoma, which casts a new light on a warning about dietary intake doses and any drug administration.

## Data availability statement

Publicly available datasets were analyzed in this study. This data can be found at: https://www.cdc.gov/nchs/nhanes/index.htm.

## Author contributions

YH: conceptualization. BX and ZL: methodology, software, formal analysis, investigation, resources, and data curation. ZY and JZ: validation, supervision, project administration, and funding acquisition. ZY, JZ, YW, YH, and BX: writing—original draft preparation, and writing—review and editing. All authors contributed to the article and approved the submitted version.

## Funding

The study was supported by the Natural Science Foundation of China (NSFC 82000877).

## Conflict of interest

The authors declare that the research was conducted in the absence of any commercial or financial relationships that could be construed as a potential conflict of interest.

## Publisher’s note

All claims expressed in this article are solely those of the authors and do not necessarily represent those of their affiliated organizations, or those of the publisher, the editors and the reviewers. Any product that may be evaluated in this article, or claim that may be made by its manufacturer, is not guaranteed or endorsed by the publisher.
